# Case Report: Suboptimal response to standard-dose asfotase alfa in perinatal hypophosphatasia indicates a need for individualized dosing

**DOI:** 10.3389/fendo.2025.1587807

**Published:** 2025-06-18

**Authors:** Danijela Petković Ramadža, Tamara Žigman, Mislav Čavka, Ivo Barić

**Affiliations:** ^1^ Department of Pediatrics, University Hospital Centre Zagreb, Zagreb, Croatia; ^2^ School of Medicine, University of Zagreb, Zagreb, Croatia; ^3^ Department of Diagnostic and Interventional Radiology, University Hospital Centre, Zagreb, Croatia

**Keywords:** alkaline phosphatase, hypophosphatasia, perinatal form, asfotase alfa, rickets, central nervous system

## Abstract

Hypophosphatasia (HPP) is a rare, inherited metabolic disorder due to a deficiency of tissue-nonspecific alkaline phosphatase, characterized by defective bone and teeth mineralization with consequent problems, including respiratory failure in severe types of HPP. Severe patients exhibit other disease-related manifestations, including neurological manifestations, which make HPP complex and difficult to manage. Enzyme replacement therapy with asfotase alfa is a disease-specific treatment for skeletal manifestations in pediatric patients. We present a patient with perinatal HPP who had a severe clinical course with respiratory insufficiency during infancy requiring a higher dose of asfotase alfa than recommended (12 mg/kg/week). After improvement of respiratory function and outgrowing the higher dose, the patient was maintained on the standard dose (6 mg/kg/week) from the age of 3 years. At 6 years of age, unexplained clinical and radiographic deterioration occurred while laboratory parameters remained normal. Following a traumatic humerus fracture that occurred after several months, asfotase alfa was increased to 10 mg/kg/week. Remarkable clinical improvement was observed and the patient regained the ability to walk unassisted 3 months after the dose correction. The rickets severity score changed from 10 to 3.5 within 14 months. No side effects from the higher drug dose have been noticed. The remaining challenge in this patient was a neurodevelopmental disorder. In conclusion, the standard dose of asfotase alfa was not sufficient to treat the skeletal manifestations of HPP in our patient, indicating that some perinatal HPP patients should be treated with higher doses to reach treatment goals. Although bone disease and patient outcomes have been improved with tailored drug doses, neurological manifestations of HPP remain challenging.

## Introduction

Hypophosphatasia (HPP) is a rare metabolic bone disease caused by low activity of tissue-nonspecific alkaline phosphatase (TNSALP) (E.C. 3.1.3.1) (UniProt ID: P05186) due to loss-of-function mutations in the *ALPL* gene (NCBI Gene: 249, Ensembl: ENSG00000162551). It can be inherited in both autosomal dominant and autosomal recessive fashions (1). TNSALP is a ubiquitous membrane-bound phosphohydrolase that hydrolyzes inorganic pyrophosphate (PPi), pyridoxal-5′-phosphate (PLP), and phosphoethanolamine (PEA). Reduced activity of TNSALP leads to substrate accumulation and subsequent metabolic alterations. The accumulation of PPi causes defective bone and teeth mineralization, while impaired hydrolysis of PLP causes vitamin B_6_ deficiency in the central nervous system (CNS), which is necessary for neurotransmitter synthesis (1). More than 450 *ALPL* variants have been described in the Johannes Kepler University *ALPL* Gene Variant Database (2). Genotype-phenotype correlation is poor with interfamilial and intrafamilial phenotypic variability (3).

According to the disease onset and severity, HPP is classified into six types. *Perinatal HPP* is the most severe and life-threatening type manifesting *in utero* with polyhydramnios, bowed and shorten long bones with osteochondral spurs, skeletal hypomineralization, and lung hypoplasia. Soon after birth, patients usually develop respiratory failure and other disease-related complications, such as pyridoxine-dependent seizures. *Benign perinatal HPP* manifests with fetal bone deformities, which resolve spontaneously, while the disease may evolve to other forms of HPP. Patients with *infantile HPP* (OMIM 241500) are asymptomatic at birth and develop signs and symptoms within six months of life, usually failure to thrive, muscle weakness, hypotonia, developmental delay, and rickets. Some patients have craniosynostosis and/or nephrocalcinosis. Seizures and respiratory failure are considered poor prognostic signs. Although milder than the perinatal form, this type has a high mortality rate of 50% if left untreated (4). *Childhood HPP* (OMIM 241510) is characterized by rickets, skeletal deformities, recurrent bone fractures, muscle weakness, and delayed growth. *Adult HPP* (OMIM 146300) presents with musculoskeletal pain, bone fractures or pseudofractures, and osteomalacia. The mildest and the most frequent form is *odontohypophosphatasia*, characterized by isolated dental problems (premature and painless loss of primary teeth and/or severe tooth decay), which are also present in other types of HPP (4).

Skeletal radiographs in pediatric patients show demineralization alongside irregular and widened metaphyses of long bones with central radiolucency called “tongues of radiolucencies”, which are characteristic of HPP (5, 6). The biochemical hallmark is persistently low alkaline phosphatase (ALP) for age and gender. As a result of impaired bone mineralization, patients usually have hypercalcemia, hyperphosphatemia, hypercalciuria, and low parathyroid hormone. Together with low ALP, useful diagnostic biomarkers are increased serum PLP and urinary PEA, while PPi analysis is not widely available. The diagnosis is usually confirmed by genetic testing, although confirmation of the causative *ALPL* gene variant is not necessary if the diagnostic criteria are met (7). Differential diagnoses of HPP include osteogenesis imperfecta, skeletal dysplasias, and diseases that cause rickets and osteomalacia, such as vitamin D-deficient and -resistant rickets, X-linked hypophosphatemia, and renal osteodystrophy. Furthermore, there are various other conditions with low ALP, for instance, hypothyroidism; hypoparathyroidism; deficiencies of zinc, magnesium, or copper; protein-energy undernutrition; coeliac disease; Wilson’s disease; excess vitamin D; delayed growth; and the use of anti-resorptive drugs (7).

Asfotase alfa is the only currently available causative treatment for bone manifestations in pediatric-onset HPP. It is a recombinant human TNSALP bound to an immunoglobulin G1 Fc domain and a deca-aspartate peptide domain, which facilitates anchorage at the calcium hydroxyapatite surface of bones (8). The first studies showed that subcutaneous injections of asfotase alfa 1 mg/kg six times per week or 2 mg/kg thrice weekly improved bone mineralization, respiratory function, and survival (9). Further clinical studies and real-world experience provided additional evidence of the safety and efficacy of the treatment in regard to radiographic parameters, respiratory function, mobility, and survival (10, 11). The registered and recommended dose by the European Medicines Agency (EMA) is 1 mg/kg six times per week or 2 mg/kg thrice weekly (8). The same dose is recommended by the Food and Drug Administration (FDA), with the possibility to increase the dose up to 9 mg/kg per week (3 mg/kg thrice weekly) if there is a lack of efficacy (12).

We present a patient with perinatal HPP in whom a higher than recommended dose of asfotase alfa was needed to reach treatment goals, both during infancy when the child was respiratory insufficient and during childhood for rickets healing.

## Case report

**Figure 1:**
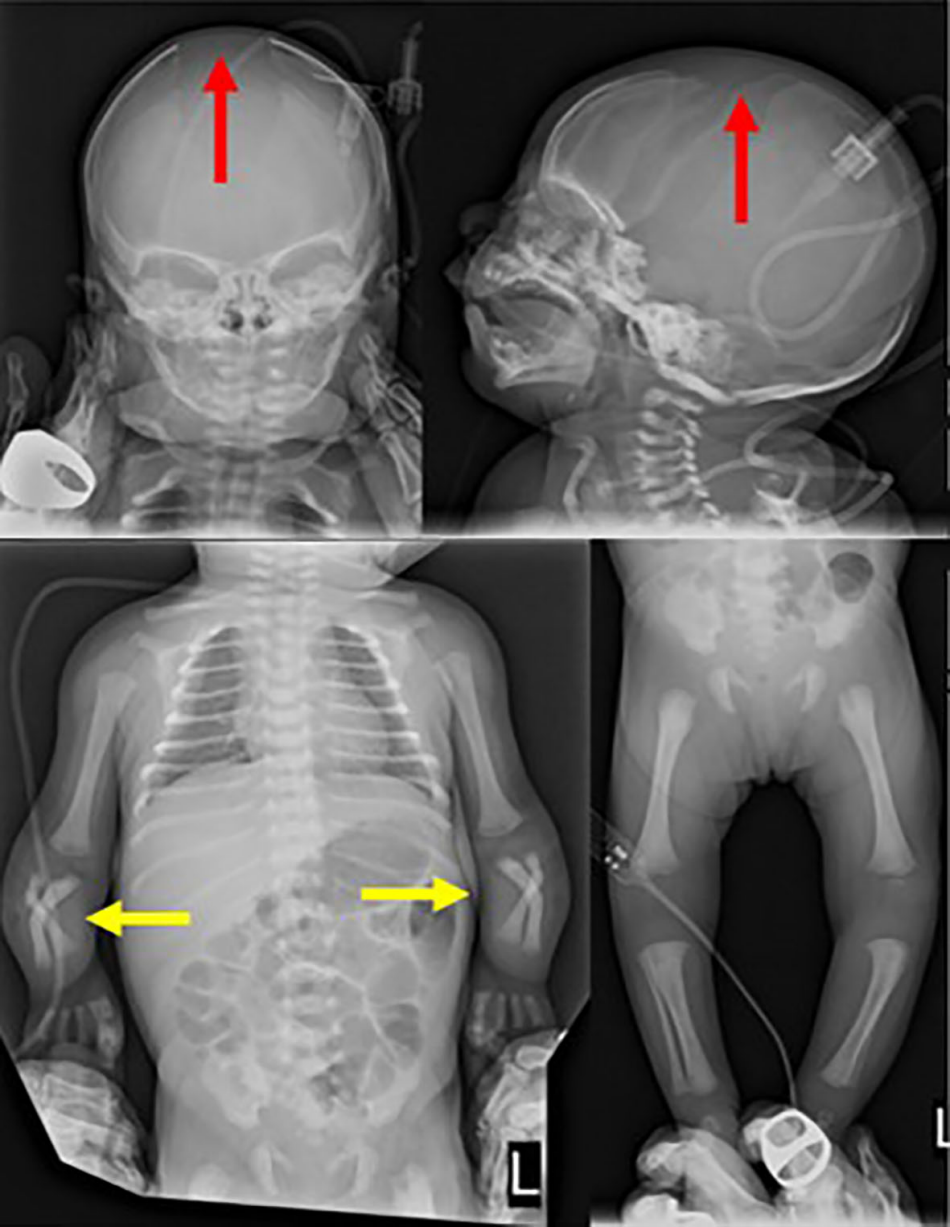
Skeletal radiographs taken on the second day of life show wide cranial sutures (red arrow) and shortened and bowed forearm bones (yellow arrow).

The female patient is the fourth child of healthy and reportedly non-consanguineous parents. Her older sister manifested neonatal seizures but had been diagnosed with infantile HPP only at the age of 5 months when she presented with failure to thrive, hypotonia, and rickets. After low ALP (4 U/L, ref. range 134–518) and hypercalcemia were measured, HPP was confirmed by genetic testing, revealing a homozygous *ALPL* mutation c.1402G>A (p.Ala468Thr). As the specific treatment was unavailable, the infant was treated symptomatically and died at the age of 8 months due to respiratory insufficiency following bilateral pneumonia (13). The parents, who are carriers, received genetic counseling but refused prenatal testing. Pregnancy was complicated with premature rupture of membranes, oligohydramnios, and threatened preterm labor at 27 weeks of gestation. There were no obvious signs of bone dysplasia on fetal ultrasound. Delivery was at 37 weeks of gestation, birth weight (BW) 2,490 g (22^nd^ percentile), birth length (BL) 46 cm (27^th^ percentile), head circumference (HC) 30.5 cm (5^th^ percentile), and Apgar scores of 9, 8, and 10. Upon birth, wide skull sutures, bowed forearms (**Figure 1**), and corneal clouding were noticed. ALP was below 5 U/L (ref. 90–273). Ophthalmological examination revealed corneal edema and ocular hypertension bilaterally, implicating congenital glaucoma. Gene analysis confirmed the same homozygous *ALPL* mutation as in her deceased sister.

Asfotase alfa treatment at a dose of 2 mg/kg subcutaneously thrice weekly started at the age of 2 months, after getting all the necessary approvals for compassionate use courtesy of Alexion Pharmaceuticals. HPP biomarkers before the treatment were as follows: PLP in plasma 51,229 pmol/L (ref. <80), PEA in plasma 43.15 µmol/L (ref. <2), and creatinine in urine 1,170 mmol/mol (ref. <50). After 1 month of treatment, the biomarker levels decreased (PLP in plasma 111 nmol/l, PEA in plasma 1.68 µmol/L, and creatinine in urine 84.2 mmol/mol), but slow respiratory deterioration and signs of rickets occurred. Therefore, 6 weeks after treatment initiation, the dose was increased to 3 mg/kg thrice weekly. Despite the dose correction, respiratory failure progressed, necessitating mechanical ventilation. Asfotase alfa dose was further increased to 4 mg/kg thrice weekly, 1 month after previous increase. The subsequent 3 months were complicated with pneumonias, atelectases, tracheobronchomalacia, and pulmonary hypertension requiring sildenafil treatment, high frequency ventilation, and tracheostomy. The patient also had other recurrent infections and sepsis. After 3 months of intensive care and high-dose asfotase alfa treatment, her condition improved and she was weaned off the ventilator.

Due to congenital glaucoma and worsening nystagmus, trabeculectomies were performed at the age of 9 months. The operations decreased intraocular pressure and improved the patient’s nystagmus and vision. She also had corneal calcifications that were non-progressive and resolved over time. Brain magnetic resonance imaging (MRI) at the age of 9 months showed no pathology. At the age of 2 years, a renal ultrasound revealed bilateral small renal calculi of size <3 mm. At that point, she also had hypercalciuria (6.7 mg/kg/day) and hyperphosphaturia (40 mg/kg/day) with normal serum calcium and phosphorus. Hydrochlorothiazide and magnesium citrate treatment led to the resolution of nephrolithiasis in 18 months. During that period, the patient had a stable course with satisfactory biochemical and radiographic parameters, thus, the asfotase alfa dose was passively decreased, allowing the patient to outgrow the dose to the standard one of 6 mg/kg weekly, at 3 years of age. Advancement in motor development was relatively satisfactory in contrast with the patient’s behavioral problems. She started to walk unassisted at the age of 3 years. Control brain MRI performed at the age of 3 years showed a normal intracranial status. Over the next 3 and a half years, the dose was corrected with her weight gain to maintain the standard one of 6 mg/kg/week.

**Figure 2:**
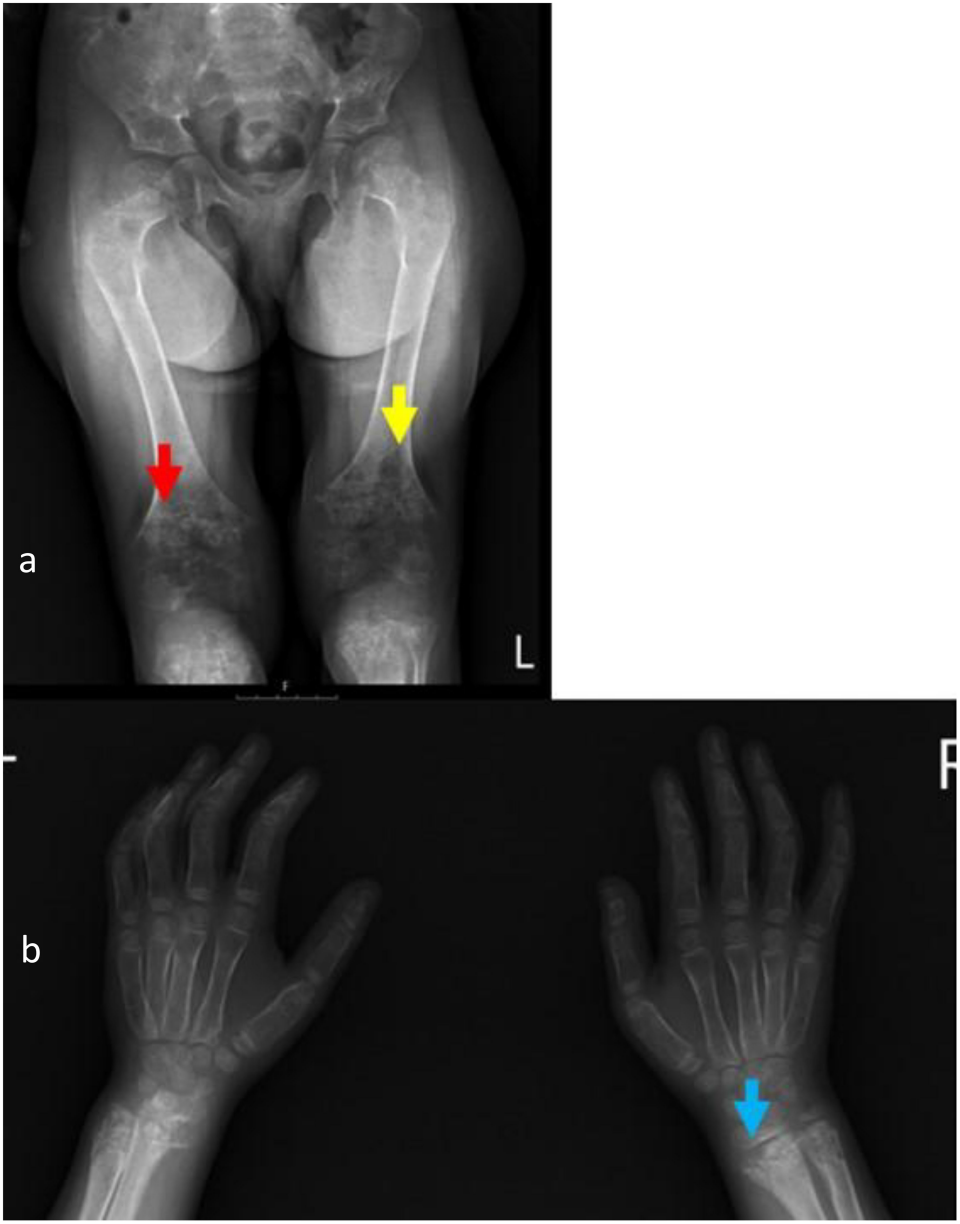
Radiographs of knees and wrists taken at the age of 6 years upon clinical deterioration show metadiaphyseal patchy focal sclerosis (red arrow) and lucencies (yellow arrow), physeal widening, irregularity of the zone of calcification, metaphyseal flaring and fraying (blue arrow), thin cortex, and demineralization of the examined bones **(a, b)**. Rickets Severity Score (RSS) = 10.

**Figure 3:**
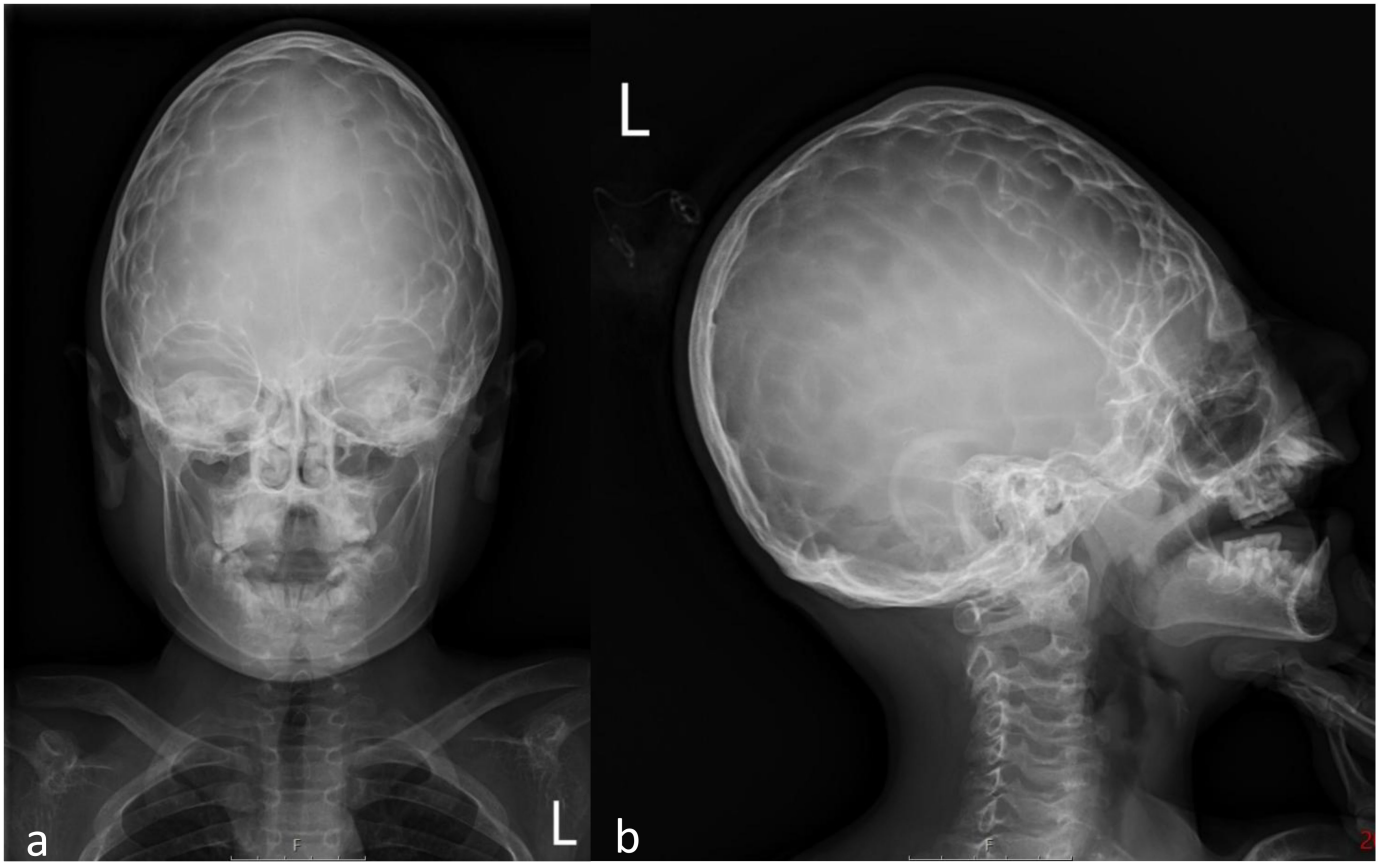
Skull radiograph taken during a follow-up 14 months after the asfotase alfa dose increase; anterior-posterior **(a)** and lateral **(b)** views show a copper-beaten appearance.

**Figure 4:**
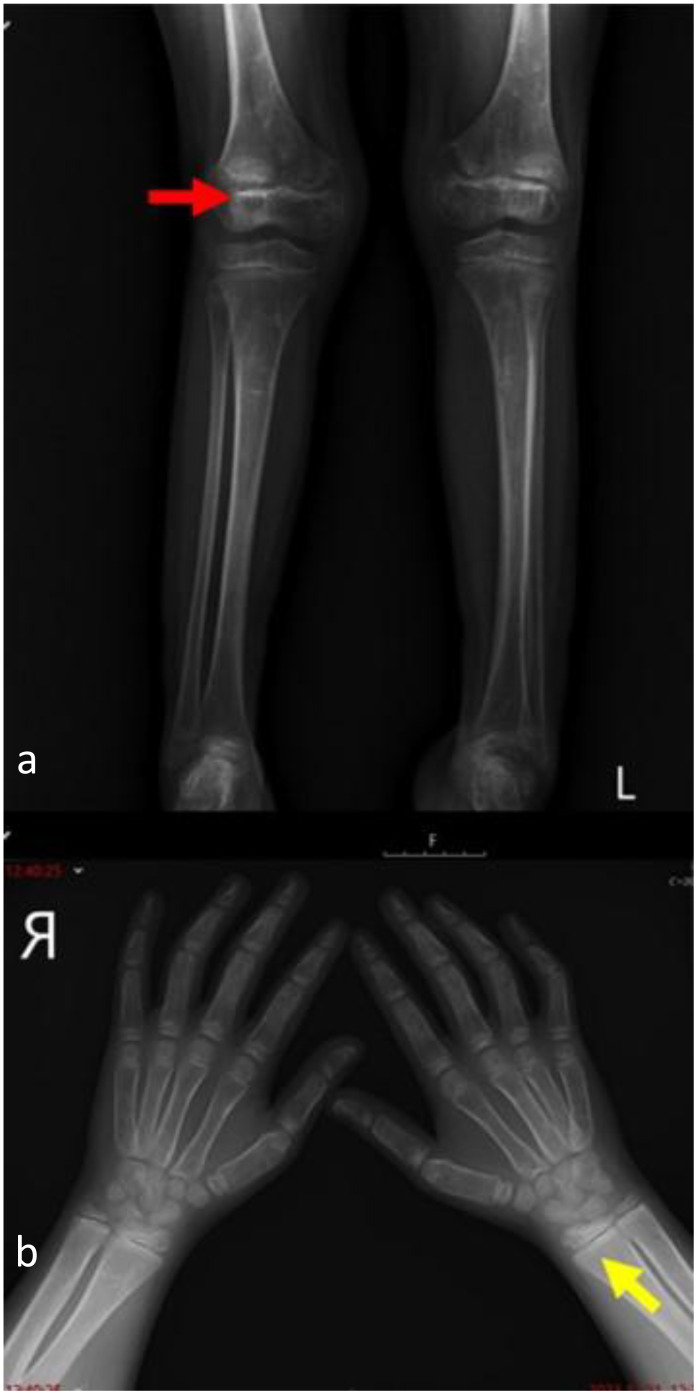
Follow-up radiographs taken 14 months after asfotase alfa dose increase to 10 mg/kg/week show improved mineralization of the lower leg bones with still-visible marginal bone irregularities at the epiphyseal regions, displaying a regressive dynamic (red arrow) **(a)**. Improved mineralization of the bones, with less-visible marginal bone irregularities at the epiphyseal regions of wrists and significant regressive dynamics (yellow arrow) **(b)**. Rickets Severity Score (RSS) = 3.5.

At the age of 6 years, clinical deterioration became evident through walking difficulties. The patient could only walk supported on bent knees. Skeletal radiographs showed significant worsening of rickets with a rickets severity score (RSS) of 10 and bone demineralization (**Figure 2**). Lumbar spine densitometry showed a bone mineral content Z-score of -6.3. Serum calcium was normal and phosphorus mildly elevated (1.93 mmoL/L, ref. range 1.2–1.8) while serum PLP/PA was within the reference range. Anti-asfotase alfa antibody testing was not available. The reason for the deterioration could not be attributed to external factors, as the patient appeared to be receiving the prescribed dose of asfotase alfa regularly and there were no significant subcutaneous changes at the injection sites that may have affected drug absorption. She was also evaluated for neurological issues and craniosynostosis due to abnormal gait. A copper-beaten skull had been present on X-rays since the age of 1 year, including the last radiogram taken at the age of 7.5 years (**Figure 3**), but neurosurgery was not previously considered because she had exhibited no other signs of intracranial hypertension, such as papilledema, vomiting, or headaches. A brain computed tomography (CT) scan was performed to reassess the new symptoms and revealed cranial bone demineralization and craniosynostosis. Upon discussion with the neurosurgeon, it was decided that craniosynostosis surgery was not indicated at that time due to her age. After the patient fractured her right humerus during a fall from a chair, the dose of asfotase alfa was increased to 10 mg/kg/week. Following the dose correction, the humerus fracture healed without complications with conservative management. After 3 months, the patient was able to extend her knees and walk without assistance. Skeletal x-rays after 6 months of treatment with the higher dose showed remarkable improvement, and further improvement was also evident after 14 months (RSS 3.5) (**Figure 4**). Lumbar spine densitometry also showed significant improvement (Z-score of -3.7). While the patient’s walking ability ameliorated, neurodevelopmental problems persisted. Neuropsychological evaluation at the age of 8 years showed severe developmental delay, undeveloped speech, and autism spectrum disorder. Another aspect of the disease is dental problems. Our patient had a premature loss of fully rooted anterior deciduous teeth, while permanent teeth manifested enamel discoloration.

## Discussion

We present a patient with perinatal HPP and a severe course in early infancy complicated by respiratory insufficiency, tracheobronchomalacia, and pulmonary hypertension that occurred despite treatment with the standard dose of asfotase alfa. Improvement occurred only after increasing the asfotase alfa dose (12 mg/kg/week). Although we had concerns about possible ectopic calcifications, especially of the cornea and kidneys, we did not observe any significant side effects during the period of high-dose treatment. Corneal calcifications were present already at the time of treatment initiation and resolved spontaneously, indicating that they were a feature of HPP rather than a side effect of the drug. Data from the Global HPP Registry showed that ectopic calcifications in the eye occur in a low percentage of patients (14). An additional ocular manifestation in our patient was congenital glaucoma, the etiology of which remains unclear. To the best of our knowledge, congenital glaucoma has not been previously reported in patients with HPP. Another issue was mild nephrolithiasis, which was resolved by hydrochlorothiazide treatment. We do not know if this was a feature of the HPP itself, or a side effect of the treatment, or a combination of both. It is important to emphasize that it is recommended that patients treated with asfotase alfa should be regularly monitored both for nephrolithiasis and ocular calcification (15).

Following clinical improvement, our patient gradually outgrew the higher drug dose and the treatment was maintained on a standard dose of 6 mg/kg/week. It was expected that the standard dose would be sufficient to maintain bone mineralization after the critical period was over and the treatment goals were achieved. However, we faced another deterioration with the recurrence of rickets, causing severe gait problems when the patient was 6 years old. Interestingly, at that time, plasma PLP was normal, which implies that this biomarker is not a reliable indicator of bone disease progression. After increasing the dose of asfotase alfa to 10 mg/kg/week, the patient’s gait improved, and follow-up radiographs showed rickets healing.

A dose of asfotase alfa of up to 3 mg/kg three times weekly is used in the clinical studies if the standard dose is not effective, and it is also recommended by the FDA for perinatal/infantile HPP (11, 12). However, there are no clear recommendations about the maintenance dose after improvement, nor about drug dose correction in case of deterioration after a period of stable clinical course. Our experience of the need for a higher drug dose is supported by a published case report of a 2-year-old patient with iHPP and recurrent fractures who had clinical and radiological improvement after an increase of asfotase alfa dose from 4.5 mg/kg/week to 12.8 mg/kg/week (16). Nevertheless, a lower than standard dose was used in an infant with HPP and a milder phenotype with satisfactory outcomes (17). This emphasizes the need for individually tailored drug doses.

Another issue was our patient’s neurological outcome, which was worse than we anticipated. It probably reflects the severity of the disease itself, although critical illness during infancy, which is a period of intensive brain development, may have been contributive. Although our patient only had turricephaly with a copper-beaten skull on X-rays as an isolated feature of increased intracranial pressure, which is considered to be a characteristic feature of HPP, we do wonder whether the neurological outcome would be better if we had opted for a surgical procedure in early childhood. The main neurological manifestations of severe hypophosphatasia are considered to be consequences of insufficient PLP dephosphorylation to pyridoxal, a vitamer that crosses the blood-brain barrier, and subsequent CNS depletion of this important cofactor of neurotransmitter synthesis (18). However, our patient never manifested seizures and serum PLP decreased to a reference range with the treatment. Moreover, TNSALP is considered to have important biological functions in neurogenesis as it is highly expressed in neuronal stem cells. The results of research studies suggest it has a significant role in the proliferation and differentiation of neural stem cells, myelination and growth of axons, and synapse maturation and maintenance (19). Hofmann and colleagues reported a patient with a complete loss of TNSALP who exhibited a severe neurological condition, characterized by cystic degeneration of the cerebral cortex and peripheral white matter, ultimately leading to progressive encephalopathy and death (20). There is another report of a patient with iHPP who, in contrast with their relatively milder skeletal demineralization, manifested refractory seizures and fatal encephalopathy despite early treatment with pyridoxine and a high dose of asfotase alfa of 12 mg/kg/week. In that patient, vitamin B_6_ vitamers were high while concentrations of biogenic amines were normal in the cerebrospinal fluid, suggesting vitamin B_6_-independent encephalopathy. Postmortem histopathology findings revealed cerebral cortical lesions in layers 2 and 3 in direct proximity to TNSALP-expressing neurons, further supporting the aforementioned theory (21). Furthermore, neuropsychiatric symptoms, although more subtle, are also present in adult patients with HPP, mainly as headaches, sleep disturbances, gait abnormalities, vertigo, depression, and anxiety (22). This suggests that neurological involvement in HPP might be complex and further research is needed to elucidate it. As asfotase alfa is a bone-targeted enzyme that does not cross the blood-brain barrier and is not expected to correct TNSALP deficiency in the CNS, there is an unmet need to provide other treatment modalities for neurological manifestations of this complex disease. Gene-based treatment or other therapeutic options that could assure delivery of TNSALP in the extra-skeletal tissues, including the brain, may be a solution for multisystemic manifestations of HPP.

## Conclusion

We present a patient with perinatal HPP who was treated with a higher than standard dose of asfotase alfa in order to reach treatment goals. This included the infancy period, when the child had a progressive life-threatening condition, and the childhood period, when a higher dose was needed for rickets healing. Our experience and data from the literature suggest that the dose of asfotase alfa should be adjusted in each patient individually in order to reach the desired treatment efficacy with minimal side effects, and that the standard dose might not be optimal for all patients with HPP. Unfortunately, neurological manifestations are not fully amenable to the currently available treatment, therefore, novel therapeutic options are needed to tackle neurological manifestations of HPP.

## Data Availability

The raw data supporting the conclusions of this article will be made available by the authors, without undue reservation.
